# Supplementary material for: Interactions between epistasis and pleiotropy in generic models of complex traits
Source: bioRxiv. 2026 Sep 30:2026.09.29.755220. Preprint. [Version 2] doi: 10.64898/2026.09.29.755220 (PMC13648569; doi:10.64898/2026.09.29.755220)
Supplement: Supplementary file 1 [file NIHPP2026.09.29.755220V2-supplement-1.pdf]

bioRxiv preprint doi: https://doi.org/10.64898/2026.09.29.755220; this version posted September 30, 2026. The copyright holder for this preprint (which was not certified by peer review) is the author/funder, who has granted bioRxiv a license to display the preprint in perpetuity. It is made available under a CC-BY 4.0 International license.

Supplemental Material

Caelan Brooks, Misha Gupta, Gautam Reddy, Michael M. Desai

Interactions between epistasis and pleiotropy in generic models of complex traits

Contents

Supplementary Figures **15**
Supplementary Text **19**
Generalization to multiple focal loci 19
Connected Architecture and optimization problem 19
Optimization problem 19
Obtaining matrices from data 19
Loss function. 20
Optimization Process 20
Fitting Hyperparameters 20
Connectedness model predictions. 21
Steady State fluctuations 21
Independent modules 21
Isolated modules 23
Homogeneous modules 24
Experimental Materials and Methods 26
Growth assay details 26
DNA Extractions for barcode sequencing 26
Sequencing Library Preparation and PCR protocols 26
Inferring mutational fitness from sequencing data 27
Background Fitness Estimation 27

Supplementary Figures

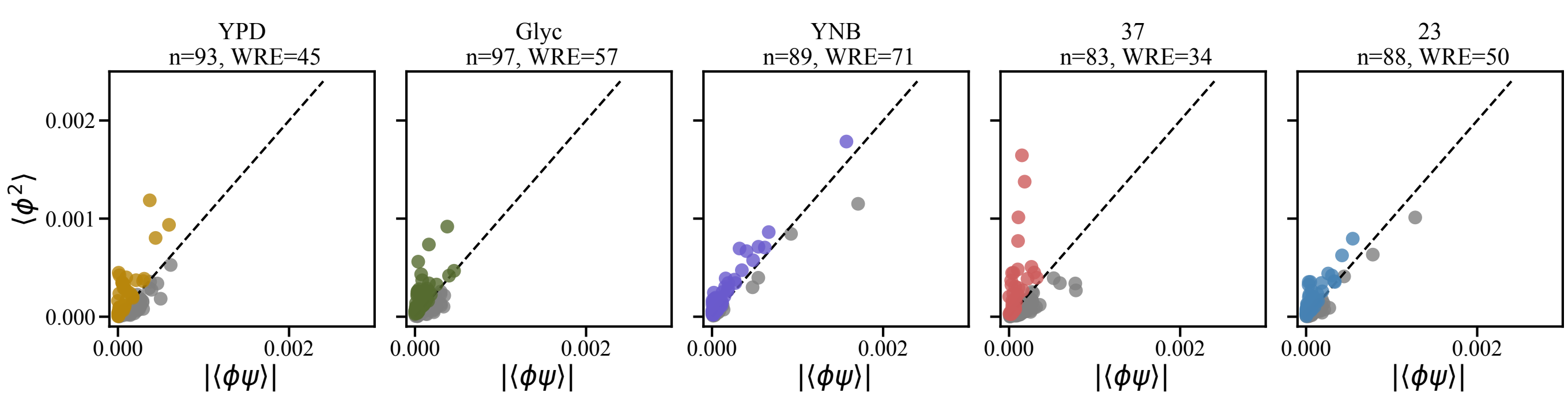

Figure S6: **Applicability of the WRE approximation in each environment**. Each point represents the values of $|\langle\phi^2_{e\alpha}\rangle|$ and $|\langle\phi_{e\alpha}\psi_{e\alpha}\rangle|$ for one focal mutation in each of our five environments. Environment identities, the total number of focal mutations, n, and the total number of these focal mutations in the WRE limit in that environment (defined as those for which $|\langle\phi^2_{\alpha e}\rangle| > |\langle\phi_{\alpha e}\psi_{\alpha e}\rangle|$ are shown at top of each panel.

bioRxiv preprint doi: https://doi.org/10.64898/2026.09.29.755220; this version posted September 30, 2026. The copyright holder for this preprint (which was not certified by peer review) is the author/funder, who has granted bioRxiv a license to display the preprint in perpetuity. It is made available under a CC-BY 4.0 International license.

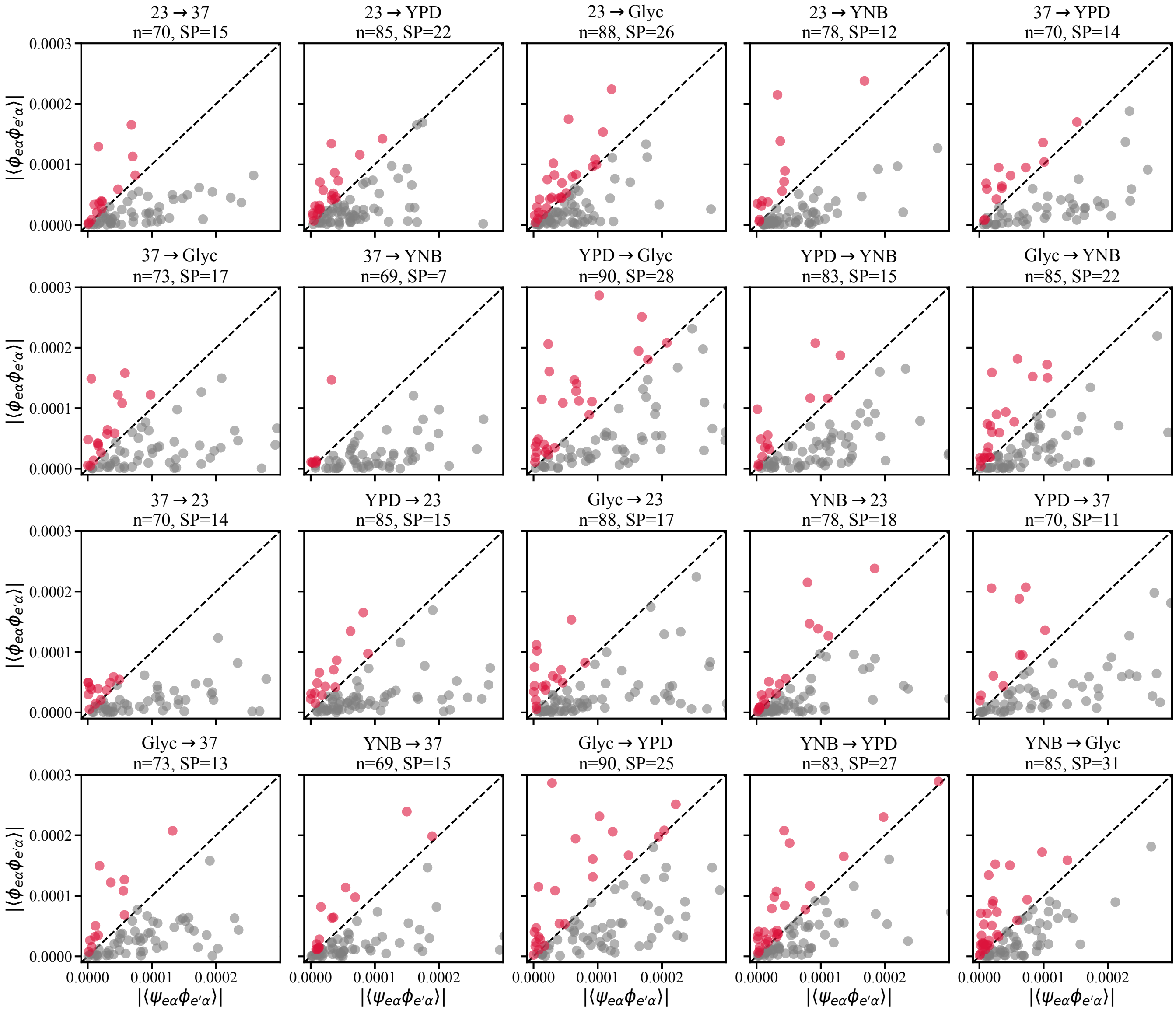

Figure S7: **Applicability of the SP approximation for each environment-pair**. Each point represents the values of $|\langle \phi_{\alpha e}\phi_{e'\alpha}\rangle|$ and $|\langle \psi_{e\alpha e}\phi_{e'\alpha}\rangle|$ for one focal mutation in each environment-pair. Environment identities, the total number of focal mutations, n, and the total number of these focal mutations in the SP limit for that environment-pair (defined as those for which $|\langle \phi_{\alpha e}\phi_{e'\alpha}\rangle| > |\langle \psi_{e\alpha e}\phi_{e'\alpha}\rangle|$) are shown at top of each panel.

bioRxiv preprint doi: https://doi.org/10.64898/2026.09.29.755220; this version posted September 30, 2026. The copyright holder for this preprint (which was not certified by peer review) is the author/funder, who has granted bioRxiv a license to display the preprint in perpetuity. It is made available under a CC-BY 4.0 International license.

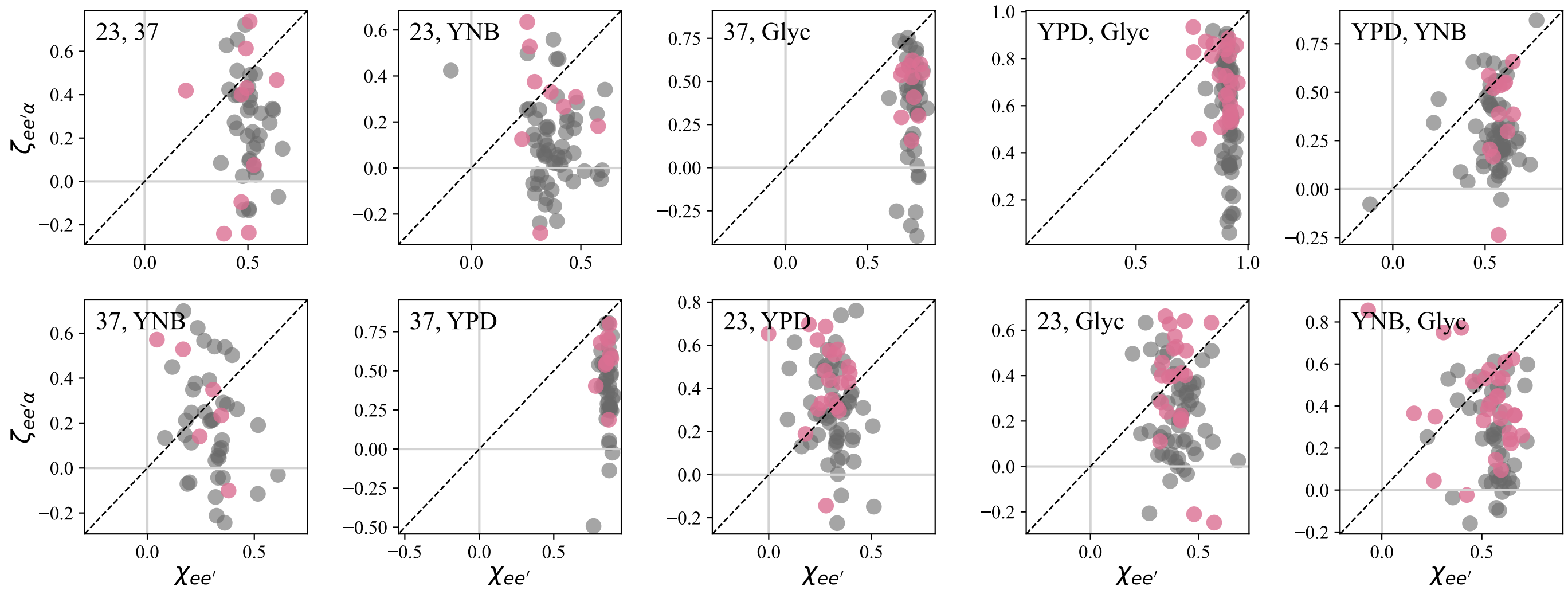

Figure S8: **Test of the fully random pleiotropy model.** Each point represents the correlation coefficient of the fitness effect of a given focal mutation across a given environment-pair, $\zeta_{ee'}$, plotted as a function of the correlation coefficient of background fitness values in that environment-pair, $\chi_{ee'}$. Focal mutations that are in the WRE and SP limits in a given environment-pair are shown in pink, while all others are shown in gray. Note that $\chi_{ee'}$ values for each focal mutation can be different due to missing data.

bioRxiv preprint doi: https://doi.org/10.64898/2026.09.29.755220; this version posted September 30, 2026. The copyright holder for this preprint (which was not certified by peer review) is the author/funder, who has granted bioRxiv a license to display the preprint in perpetuity. It is made available under a CC-BY 4.0 International license.

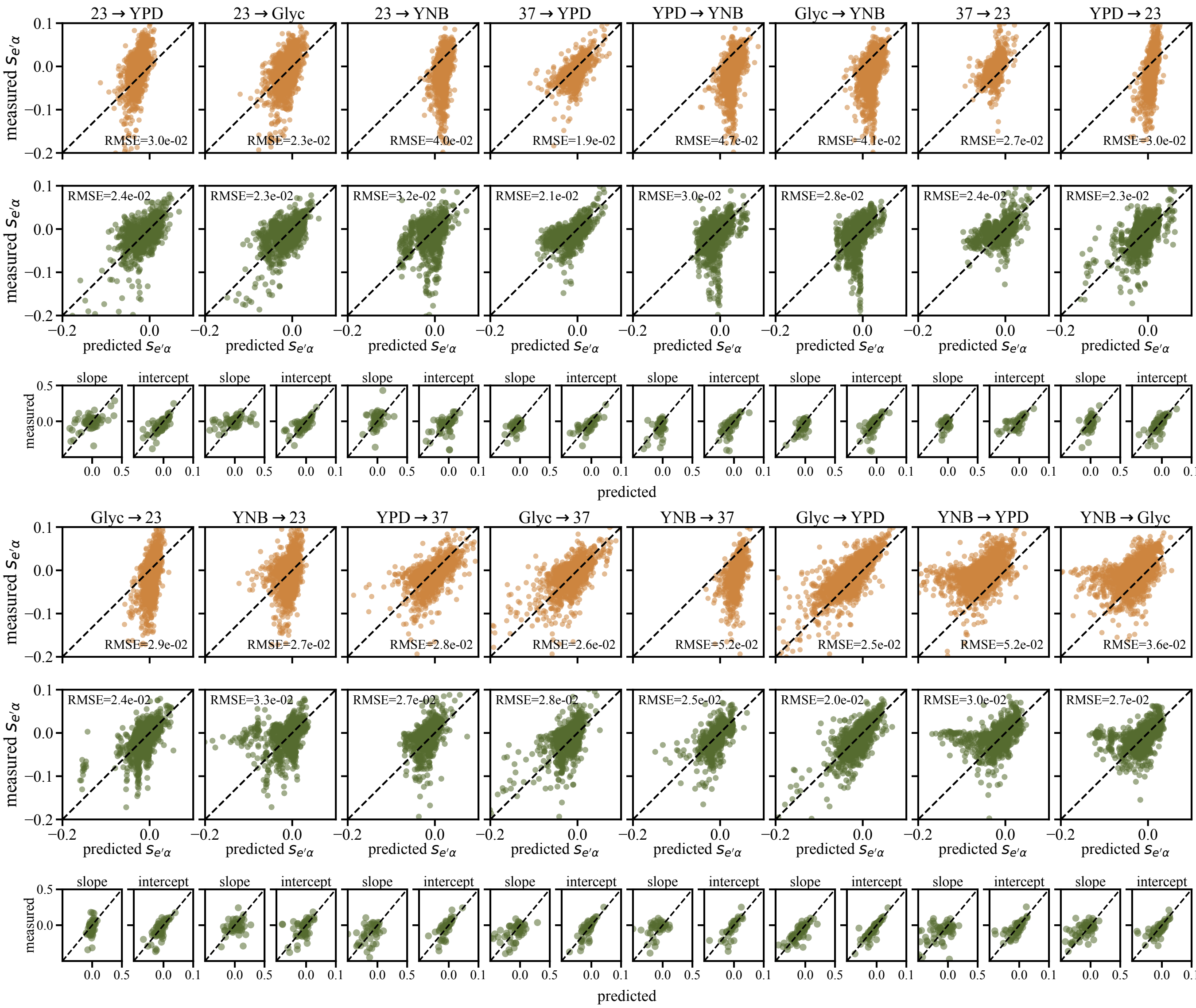

Figure S9: **Test of the criss-cross environment prediction in all environment-pairs.** Yellow plots show test of the baseline prediction that the relationship between fitness effects of focal mutations is identical to the relationship between background fitnesses. Large green plots show test of the criss-cross environmental prediction from our generic null model. Small green plots show predicted slopes and intercepts of the relationship between $s_{e'\alpha}$ and $s_{e\alpha}$ from our generic null model.

bioRxiv preprint doi: https://doi.org/10.64898/2026.09.29.755220; this version posted September 30, 2026. The copyright holder for this preprint (which was not certified by peer review) is the author/funder, who has granted bioRxiv a license to display the preprint in perpetuity. It is made available under a CC-BY 4.0 International license.

Supplementary Text

Generalization to multiple focal loci

Throughout the text we have focused on the case of a single focal locus ($F = 1$). However, the data analyzed includes focal mutations all made on the same set of background genotypes. We now consider how our results can be generalized to the case where we measure the fitness effect of multiple ($F > 1$) focal loci (as before, in E different environments across a large number of genetic backgrounds that vary across L background loci). Because we do not consider the effects of making multiple focal mutations simultaneously, we can treat each focal locus separately. In other words, for focal locus γ, we can write the fitness in environment e as

$$y_e(\vec{x}, x_\gamma) = \tilde{f}_{e\gamma} + \psi_{e\gamma}(\vec{x}) + x_\gamma \left[f_{e\gamma} + \phi_{e\gamma}(\vec{x})\right]. \tag{S29}$$

However, it is important to note that all terms in this equation now depend on which focal locus we are considering, and include contributions from terms involving all the other focal loci.

To see this, consider the case of two focal loci, which we refer to as x_α and x_β. We have

$$\begin{aligned} y_e(\vec{x}, x_\alpha, x_\beta) \quad = \quad & \tilde{f}_e + \psi_e(\vec{x}) + x_\alpha \left[f_{e\alpha} + \phi_{e\alpha}(\vec{x})\right] + \\ & x_\beta \left[f_{e\beta} + \phi_\beta(\vec{x})\right] + x_\alpha x_\beta \left[f_{e\alpha\beta} + \phi_{e\alpha\beta}(\vec{x})\right], \end{aligned} \tag{S30}$$

where ψ_e, $\phi_{e\alpha}$, and $\phi_{e\beta}$ are as defined in Eq. (2) and Eq. (3) respectively, and $\phi_{e\alpha\beta}$ involves all f terms that depend on both focal loci and one or more background loci. We can immediately see that if we consider only focal locus x_α (and hence set $x_\beta = -1$ throughout), we can define $\tilde{f}_{e\alpha} = \tilde{f}_e - f_{e\beta}$ and $\psi_{e\alpha} = \psi_e - \phi_{e\beta}$, and redefine $f_{e\alpha} \to f_{e\alpha} - f_{e\alpha\beta}$ and $\phi_{e\alpha} \to \phi_{e\alpha} - \phi_{e\alpha\beta}$, in order to recover Eq. (S29). We can implement analogous redefinitions if we only consider focal locus x_β. All of our results from above then apply to each focal locus, with the appropriately defined and redefined quantities.

We can implement a similar set of definitions and redefinitions for larger numbers of focal loci. We note that, using these definitions, we have a total of $2FE + 2FEn$ parameters: FE terms $\tilde{f}_{e\gamma}$, FE terms $f_{e\gamma}$, FEn terms $\psi_{e\gamma}$, and FEn terms $\phi_{e\gamma}$. This is much more than the total number of data points ($En + FEn$). However, we also have a corresponding set of constraints (e.g. $y_e(\vec{x}, x_\gamma = -1)$ must be independent of which focal locus we consider). Using these constraints, it is straightforward to compute all of the parameters relevant for each focal locus directly from the data. We can then compute all of the relevant variances and covariances (e.g. $\langle \psi^2 \rangle$, $\langle \phi^2 \rangle$, $\langle \phi_e \phi_{e'} \rangle$, etc), and use these to compute expected patterns of global epistasis and pleiotropy for each focal locus as described above.

Connected Architecture and optimization problem

In this section, we describe the process used to fit parameters in the connectedness model from the background fitness and fitness effect data.

Optimization problem

Eq. (18) in the main text outlines the relationship between measured quantities (i.e. the variances and covariances of $\psi's$), and $\phi's$, and the model parameters which describe interaction structure (i.e. the $w's$ and $v's$). Our goal here is to infer the latter from the former. It is useful to compile these variances and covariances, along with the isolated additive effects, into matrices that can be utilized in an optimization framework. We tabulate them here:

$$\mathbf{F_e} = \begin{pmatrix} f_{e\alpha} \\ f_{e\beta} \\ \vdots \end{pmatrix}, \qquad \mathbf{C_{ee}} = \begin{pmatrix} \langle \psi_e^2 \rangle & \langle \psi_e \psi_{e'} \rangle & \cdots \\ \langle \psi_{e'} \psi_e \rangle & \langle \psi_{e'}^2 \rangle & \cdots \\ \vdots & \vdots & \ddots \end{pmatrix}, \qquad \mathbf{T}_\alpha = \begin{pmatrix} \langle \phi_e^2 \rangle & \langle \phi_e \phi_{e'} \rangle & \cdots \\ \langle \phi_{e'} \phi_e \rangle & \langle \phi_{e'}^2 \rangle & \cdots \\ \vdots & \vdots & \ddots \end{pmatrix}.$$

Here the $\mathbf{F_e}$ vector contains the isolated additive effects of the focal loci, $\mathbf{C_{ee}}$ encodes the correlation between the background loci interactions between all environments, and $\mathbf{T}_\alpha$ represents the correlation between interaction terms of a focal locus in different environments (note there is one matrix $\mathbf{T}_\alpha$ for each of the F focal loci).

Obtaining matrices from data

These matrices consist of $E \times F$ values of $f_{e\alpha}$, E values of ψ_e, and $F \times E$ values $\phi_{e\alpha}$, each of which is found directly from data. We start with the generalized multi-locus fitness description from Eq. (S29) in the main text,

$$y_e(\vec{x}, x_\alpha) = \tilde{f}_{e\alpha} + \psi_{e\alpha}(\vec{x}) + x_\alpha[f_{e\alpha} + \phi_{e\alpha}(\vec{x})].$$

At each focal locus, we have the fitness values with and without the focal mutation,

$$\begin{aligned} y_e^-(\vec{x}) &= \tilde{f}_{e\alpha} + \psi_{e\alpha}(\vec{x}) - f_{e\alpha} - \phi_{e\alpha}(\vec{x}), \\ y_e^+(\vec{x}) &= \tilde{f}_{e\alpha} + \psi_{e\alpha}(\vec{x}) + f_{e\alpha} + \phi_{e\alpha}(\vec{x}). \end{aligned}$$

bioRxiv preprint doi: https://doi.org/10.64898/2026.09.29.755220; this version posted September 30, 2026. The copyright holder for this preprint (which was not certified by peer review) is the author/funder, who has granted bioRxiv a license to display the preprint in perpetuity. It is made available under a CC-BY 4.0 International license.

The isolated additive effects of each focal mutation are found from the means over all background genotypes, $f_{e\alpha} = \left(\overline{y^e_+(\vec{x})} - \overline{y^e_-(\vec{x})}\right)/2$. Subtracting off the means we can find the $\psi's$ and $\phi's$,

$$\psi_{e\alpha}(\vec{x}) = \frac{\left(y_e^+(\vec{x}) - \overline{y_e^+(\vec{x})}\right) + \left(y_e^-(\vec{x}) - \overline{y_e^-(\vec{x})}\right)}{2},$$
$$\phi_{e\alpha}(\vec{x}) = \frac{\left(y_e^+(\vec{x}) - \overline{y_e^+(\vec{x})}\right) - \left(y_e^-(\vec{x}) - \overline{y_e^-(\vec{x})}\right)}{2}.$$

This gives us $E \times F$ values of $\psi_{e\alpha}(\vec{x})$ and $\phi_{e\alpha}(\vec{x})$. However, each background $y_e^-(\vec{x})$ is the same for all focal loci mutations. We want to have one description of the background fitness variance for each environment. Therefore, to find ψ_e from each $\psi_{e\alpha}$ term, we change basis using

$$\psi_e = \psi_{e\alpha} + \sum_{\beta \neq \alpha} \phi_{e\beta}. \tag{S31}$$

We find that the ψ_e values obtained from different $\psi_{e\alpha}$ values are in agreement. Our goal once fitting the model is to find the relationship between $s_{e'\alpha}$ and y_e from the model, which is composed of components of each matrix.

Loss function.

Given the previous definitions, we aim to minimize the following loss function:

$$L(\mathbf{W},\mathbf{V},\mathbf{M}) = ||\mathbf{F_e} - \mathbf{WM}||_2^2 + \lambda_C ||\mathbf{C_{ee}} - \mathbf{WW}^\top||_2^2 + \lambda_T \sum_\alpha ||\mathbf{T}_\alpha - \mathbf{WD}_\alpha \mathbf{W}^\top||_2^2, \tag{S32}$$

where $\mathbf{W}$ has matrix elements $w_{e\mu}$, $\mathbf{M}$ has elements $g_{\mu\alpha}$, $\mathbf{D}_\alpha = \mathrm{diag}(\mathbf{V}_\alpha)$ and $\mathbf{V}$ contains $v_{\mu\alpha}$ values for each locus, for all modules. Specifically, $\mathbf{F_e} \in \mathbb{R}^{E\times F}$, $\mathbf{W} \in \mathbb{R}^{E\times K}$, $\mathbf{M} \in \mathbb{R}^{K\times F}$, $\mathbf{C_{ee}} \in \mathbb{R}^{E\times E}$, $\mathbf{T}_\alpha \in \mathbb{R}^{E\times E}$ for $\alpha = 1, .., F$ and $\mathbf{V} \in \mathbb{R}^{F\times K}$. The hyper-parameters, λ_C, λ_T, and K (the number of modules), are fit according to the description in the following sections.

Optimization Process

We go through the following optimization schedule to obtain $\mathbf{W}$, $\mathbf{V}$, and $\mathbf{M}$:

1. Least squares regression to update $\mathbf{M}$.
2. Non-negative least squares regression to update $v_{\mu\alpha}$'s which feed into $\mathbf{V}$.
3. Conduct gradient steps on $\mathbf{W}$.

Step 3 requires identifying the gradient of the loss function with respect to $\mathbf{W}$. We can do this analytically by taking the derivative w.r.t. $\mathbf{W}$ of combinations of matrices,

$$\nabla_{\mathbf{W}} L(\mathbf{W},\mathbf{V},\mathbf{M}) = 2(\mathbf{WM} - \mathbf{F_e})\mathbf{M}^T + 4\lambda_C(\mathbf{WW}^T - \mathbf{C_{ee}})\mathbf{W} + 4\lambda_T \sum_\alpha (\mathbf{WD}_\alpha \mathbf{W}^T - \mathbf{T}_\alpha)\mathbf{WD}_\alpha. \tag{S33}$$

We then use this analytic gradient to take steps which change $\mathbf{W}$ with the goal of minimizing loss,

$$\mathbf{W} \to \mathbf{W} - \eta \nabla_{\mathbf{W}} L(\mathbf{W},\mathbf{V},\mathbf{M}).$$

Fitting Hyperparameters

The loss function in Eq. (S32) establishes two sets of parameters: the model parameters $\mathbf{V}$, $\mathbf{W}$, and $\mathbf{M}$, and hyper-parameters λ_C, λ_T, and the number of modules, K, where K is implicit in the dimension of matrices $\mathbf{W}$ and $\mathbf{M}$. To determine parameters we first identify the collection of mutations which have measurements on at least 10 backgrounds in all environments. We then split the effects of each mutation into train/validation/test subsets (80%, 10%, 10% respectively) multiple times (the effects of the same mutations on different backgrounds are sorted into different subsets). For each split, we build numerical targets (F, C, T) and then fit the model using a grid of different hyperparameter combinations (K,λ_C, λ_T) which control the model complexity and regularization strength. The model's mean squared error (MSE) on the validation data is used to rank configurations. After averaging validation results across repeated random splits, we select the best hyperparameters, retrain the model, evaluate it on held-out test data, and report mean ± standard-deviation MSEs for the three components ($\mathbf{F_e}$, $\mathbf{C_{ee}}$, $\mathbf{T}_\alpha$). We find that the hyperparameters, $\lambda_C = 0.01$, $\lambda_T = 10^6$, and $K = 4$ perform the best on the validation data set. Five different splits were used to determine the best performing combination.

bioRxiv preprint doi: https://doi.org/10.64898/2026.09.29.755220; this version posted September 30, 2026. The copyright holder for this preprint (which was not certified by peer review) is the author/funder, who has granted bioRxiv a license to display the preprint in perpetuity. It is made available under a CC-BY 4.0 International license.

Connectedness model predictions.

In order to predict the slope and intercept of $\langle s_{e'}|y_e\rangle$ from the outputs of the connectedness model architecture, we must change basis from $\psi_e \to \psi_{e\alpha}$. More specifically, due to the form of the output of the model we must find $\langle \psi_{e\alpha}^2\rangle$ using $\langle \psi_e^2\rangle$. From Eq. (S31) we have

$$\langle \psi_e^2\rangle = \left\langle \left(\psi_{e\alpha} + \sum_{\beta\neq\alpha}\phi_\beta(\vec{x})\right)^2\right\rangle. \tag{S34}$$

We can therefore write $\langle \psi_{e\alpha}^2\rangle$ as:

$$\langle \psi_{e\alpha}^2\rangle = \langle \psi_e^2\rangle - \sum_{\beta\neq\alpha}\langle\phi_\beta^2\rangle - 2\sum_{\beta\neq\alpha}\langle \psi_{e\alpha}\phi_\beta(\vec{x})\rangle - \sum_{\beta\neq\alpha}\sum_{\substack{\gamma\neq\alpha\\ \gamma\neq\beta}}\langle\phi_\beta\phi_\gamma\rangle. \tag{S35}$$

In the WRE limit, terms of the form $\langle \psi_{e\alpha}\phi_\beta(\vec{x})\rangle$ should be much smaller than the $\langle\phi_\beta^2\rangle$ terms. The last term in the expression represents interactions which are shared by focal loci, which we also expect to be small compared to $\langle\phi_\beta^2\rangle$. Under these assumptions we change basis using the equation $\langle \psi_{e\alpha}^2\rangle = \langle \psi_e^2\rangle - \sum_{\beta\neq\alpha}\langle\phi_\beta^2\rangle$. After doing so we can write the predictions for the slope and intercept in the form of Eq. (6).

Steady State fluctuations

We investigate three different model architectures with their own prescriptions for the relationship between loci and modules. Here we set $\mu = 1$ and $\mu' = 2$ as well as $w_{e\mu} = \beta_1$, $w_{e\mu'} = \beta_2$, $w_{e'\mu} = \alpha_1$, and $w_{e'\mu'} = \alpha_2$ for ease of understanding in the following calculations.

In the independent case, the level of participation of a locus in different modules is drawn independently. If the modules are isolated, a given locus only participates in one module, and in the homogeneous modules case, loci will have the same level of participation in each module. In this section, using the JDFE's previously calculated, we determine the expectations for the module 1 output when selecting in environment e, $\langle\sigma_\mu\rangle_e$, as well as the three other cases: the output in module 1 when selecting in environment e', $\langle\sigma_\mu\rangle_{e'}$, the module 2 output when selecting in environment e, $\langle\sigma_{\mu'}\rangle_e$, and the module 2 output when selecting in environment e', $\langle\sigma_{\mu'}\rangle_{e'}$.

Independent modules

In the case of modules in which the loci choose their degree of participation independently for each module (μ_{im} drawn independently), we want to solve the integral,

$$\langle\sigma_1\rangle_e = 2\int_{-\infty}^{\infty}\int_{-\infty}^{\infty} d\sigma_2 d\sigma_1 \sigma_1(\beta_1\sigma_1+\beta_2\sigma_2)\Theta(\beta_1\sigma_1+\beta_2\sigma_2)\rho(\sigma_1|h_1)\rho(\sigma_2|h_2). \tag{S36}$$

Here the heaviside function allows for only positive mutations to be selected based on the fixation probability $p_{\text{fix}} = 2s_e = 2(\beta_1\sigma_1 + \beta_2\sigma_2)$. We can write this as

$$\langle\sigma_1\rangle_e = 2\int_{-\infty}^{\infty}\frac{d\lambda}{2\pi i(\lambda - i\epsilon)}\int_{-\infty}^{\infty}\int_{-\infty}^{\infty} d\sigma_2 d\sigma_1\sigma_1(\beta_1\sigma_1+\beta_2\sigma_2)e^{i\lambda(\beta_1\sigma_1+\beta_2\sigma_2)}\rho(\sigma_1|h_1)\rho(\sigma_2|h_2), \tag{S37}$$

where

$$\rho(\sigma_m|h_m) = \frac{\nu_0^{-1}}{2\sqrt{2\nu_0^{-1}+h_m^2}}e^{-\sigma_m h_m/2 - |\sigma_m|\sqrt{2\nu_0^{-1}+h_m^2}/2}. \tag{S38}$$

We first rescale with $\sigma'_m = \beta_m\sigma_m$,

$$= 2\beta^{-1}\int_{-\infty}^{\infty}\frac{d\lambda}{2\pi i(\lambda - i\epsilon)}\int_{-\infty}^{\infty}\int_{-\infty}^{\infty} d\sigma'_2 d\sigma'_1\sigma'_1(\sigma'_1+\sigma'_2)e^{i\lambda(\sigma'_1+\sigma'_2)}\rho(\sigma'_1|h_1)\rho(\sigma'_2|h_2), \tag{S39}$$

and plugging in $\sigma_m = \sigma'_m/\beta_m$ to Eq. (S38) we get

$$\rho(\sigma'_m|M_m) = \frac{\nu_0^{-1}}{2\sqrt{2\nu_0^{-1}+h_m^2}}e^{-|\sigma'_m|\zeta_m^{\pm}},$$

bioRxiv preprint doi: https://doi.org/10.64898/2026.09.29.755220; this version posted September 30, 2026. The copyright holder for this preprint (which was not certified by peer review) is the author/funder, who has granted bioRxiv a license to display the preprint in perpetuity. It is made available under a CC-BY 4.0 International license.

where $\zeta_m^{\pm} = \left(\sqrt{2\nu_0^{-1} + h_m^2} \pm h_m\hat{\beta}_m\right)/2|\beta_m|$. More specifically, ζ_m^+ is the scaling when $\sigma_m' > 0$ and ζ_m^- is the scaling when $\sigma_m' < 0$ and $\beta_m = |\beta_m|\hat{\beta}_m$. Returning to Eq. (S39) we can expand the last two integrals with

$$\int_{-\infty}^{\infty}\int_{-\infty}^{\infty} d\sigma_2' d\sigma_1' \sigma_1'(\sigma_1' + \sigma_2') e^{i\lambda(\sigma_1'+\sigma_2')} \rho(\sigma_1'|h_1)\rho(\sigma_2'|h_2)$$
$$= \int_{-\infty}^{\infty} d\sigma_2' e^{i\lambda\sigma_2'}\rho(\sigma_2'|h_2) \int_{-\infty}^{\infty} d\sigma_1'\sigma_1'(\sigma_1'+\sigma_2') e^{i\lambda\sigma_1'}\rho(\sigma_1'|h_1)$$
$$= \int_{-\infty}^{\infty} d\sigma_2' e^{i\lambda\sigma_2'}\rho(\sigma_2'|h_2) \left(\int_{-\infty}^{\infty} d\sigma_1'\sigma_1'^2 e^{i\lambda\sigma_1'}\rho(\sigma_1'|h_1) + \sigma_2' \int_{-\infty}^{\infty} d\sigma_1'\sigma_1' e^{i\lambda\sigma_1'}\rho(\sigma_1'|h_1)\right).$$

Expanding further we get

$$= \int_{-\infty}^{\infty} d\sigma_2' e^{i\lambda\sigma_2'}\rho(\sigma_2'|h_2) \int_{-\infty}^{\infty} d\sigma_1'\sigma_1'^2 e^{i\lambda\sigma_1'}\rho(\sigma_1'|h_1) + \int_{-\infty}^{\infty} d\sigma_2'\sigma_2' e^{i\lambda\sigma_2'}\rho(\sigma_2'|h_2) \int_{-\infty}^{\infty} d\sigma_1'\sigma_1' e^{i\lambda\sigma_1'}\rho(\sigma_1'|h_1) \tag{S40}$$

Here it is useful to move into fourier space and use the characteristic functions of the DFE's. Using the following identities,

$$\tilde{\rho}(\lambda) = \int_{-\infty}^{\infty} e^{i\lambda x}\rho(x)\,dx, \qquad \frac{d\tilde{\rho}(\lambda)}{d\lambda} = \int_{-\infty}^{\infty} ix\,e^{i\lambda x}\rho(x)\,dx, \qquad \frac{d^2\tilde{\rho}(\lambda)}{d\lambda^2} = \int_{-\infty}^{\infty} -x^2 e^{i\lambda x}\rho(x)\,dx.$$

Eq. (S40) can be written as

$$= -\left(\tilde{\rho}_2 \frac{d^2\tilde{\rho}_1}{d\lambda^2} + \frac{d\tilde{\rho}_1}{d\lambda}\frac{d\tilde{\rho}_2}{d\lambda}\right) = -\frac{d}{d\lambda}\left(\tilde{\rho}_2 \frac{d\tilde{\rho}_1}{d\lambda}\right)$$

and Eq. (S39) becomes

$$\langle\sigma_1\rangle_s = -2\beta_1^{-1} \int_{-\infty}^{\infty} \frac{d\lambda}{2\pi i(\lambda - i\epsilon)} \frac{d}{d\lambda}\left(\tilde{\rho}_2 \frac{d\tilde{\rho}_1}{d\lambda}\right) \tag{S41}$$
$$= -2\beta_1^{-1} \int_{-\infty}^{\infty} \frac{d\lambda}{2\pi i(\lambda - i\epsilon)^2} \tilde{\rho}_2 \frac{d\tilde{\rho}_1}{d\lambda}. \tag{S42}$$

For an exponential distribution, the characteristic function is:

$$\tilde{\rho}(\lambda, \zeta_m^+) = \int_0^{\infty} e^{i\lambda x}\zeta_m^+ e^{-\zeta_m^+ x} = \frac{\zeta_m^+}{\zeta_m^+ - i\lambda} = \frac{i\zeta_m^+}{\lambda + i\zeta_m^+},$$
$$\tilde{\rho}(\lambda, \zeta_m^-) = \int_{-\infty}^{0} e^{i\lambda x}\zeta_m^- e^{-\zeta_m^- x} = \frac{\zeta_m^-}{\zeta_m^- + i\lambda} = -\frac{i\zeta_m^-}{\lambda - i\zeta_m^-}.$$

If we want $\tilde{\rho}(\lambda)$ over all values of σ_m' we are summing over the random variables

$$\int_{-\infty}^{0} d\sigma_m' \rho(\sigma_m'; \zeta_m^-) + \int_0^{\infty} d\sigma_m' \rho(\sigma_m'; \zeta_m^+).$$

We therefore have to multiply the characteristic functions to get the general form,

$$\tilde{\rho}(\lambda) = \frac{\zeta_m^- \zeta_m^+}{(\lambda + i\zeta_m^+)(\lambda - \zeta_m^-)}.$$

Returning to Eqn. S42 we now have

$$\langle\sigma_1\rangle_s = 2\beta_1^{-1} \int_{-\infty}^{\infty} \frac{d\lambda}{2\pi i(\lambda - i\epsilon)^2} \frac{\zeta_1^+\zeta_1^-\zeta_2^+\zeta_2^-}{(\lambda + i\zeta_1^+)(\lambda - i\zeta_1^-)(\lambda + i\zeta_2^+)(\lambda - i\zeta_2^-)} \left(\frac{1}{\lambda + i\zeta_1^+} + \frac{1}{\lambda - i\zeta_1^-}\right), \tag{S43}$$

and we want to evaluate the two integrals

$$= 2\beta_1^{-1} \int_{-\infty}^{\infty} \frac{d\lambda}{2\pi i(\lambda - i\epsilon)^2} \frac{\zeta_1^+\zeta_1^-\zeta_2^+\zeta_2^-}{(\lambda + i\zeta_1^+)^2(\lambda - i\zeta_1^-)(\lambda + i\zeta_2^+)(\lambda - i\zeta_2^-)} \tag{S44}$$
$$2\beta_1^{-1} \int_{-\infty}^{\infty} \frac{d\lambda}{2\pi i(\lambda - i\epsilon)^2} \frac{\zeta_1^+\zeta_1^-\zeta_2^+\zeta_2^-}{(\lambda + i\zeta_1^+)(\lambda - i\zeta_1^-)^2(\lambda + i\zeta_2^+)(\lambda - i\zeta_2^-)}. \tag{S45}$$

We close the contour in the lower half of the complex plane to avoid dealing with two double poles. The poles we enclose can be addressed by finding their residues. For the first integral we have:

$$f_1(\lambda) = \left(\frac{1}{2\pi i(\lambda - i\epsilon)^2}\right)\left(\frac{1}{(\lambda + i\zeta_1^+)^2(\lambda - i\zeta_1^-)(\lambda + i\zeta_2^+)(\lambda - i\zeta_2^-)}\right),$$

bioRxiv preprint doi: https://doi.org/10.64898/2026.09.29.755220; this version posted September 30, 2026. The copyright holder for this preprint (which was not certified by peer review) is the author/funder, who has granted bioRxiv a license to display the preprint in perpetuity. It is made available under a CC-BY 4.0 International license.

and

$$\oint_\gamma f_1(\lambda)d\lambda = 2\pi i \left[\mathrm{Res}(f_1, -i\zeta_1^+) + \mathrm{Res}(f_1, -i\zeta_2^+)\right].$$

Looking at the simpler pole first:

$$\mathrm{Res}(f_1, -i\zeta_2^+) = \frac{1}{2\pi i}\left(\frac{-1}{(\zeta_2^+)^2(\zeta_2^+ + \zeta_1^-)(\zeta_2^+ - \zeta_1^+)^2(\zeta_2^+ + \zeta_2^-)}\right).$$

And for the repeated pole we have:

$$\mathrm{Res}(f_1, -i\zeta_2^-) = \frac{1}{2\pi i}\frac{1}{(\zeta_1^+)^2(\zeta_1^+ + \zeta_1^-)(\zeta_1^+ - \zeta_2^+)(\zeta_1^+ + \zeta_2^-)}\left[\frac{2}{\zeta_1^+} + \frac{1}{\zeta_1^+ + \zeta_2^-} + \frac{1}{\zeta_1^+ - \zeta_2^+} + \frac{1}{\zeta_1^+ + \zeta_1^-}\right].$$

From here we can follow out similar procedures to find the result of the entire integral.

$$\langle \sigma_1 \rangle_s = 2\beta_1^{-1}\zeta_1^+\zeta_1^-\zeta_2^+\zeta_2^-(I_1 + I_2 + I_3 + I_4), \tag{S46}$$

where

$$\begin{aligned}
I_1 &= \frac{1}{(\zeta_1^+)^2(\zeta_1^+ + \zeta_1^-)^2(\zeta_1^+ - \zeta_2^+)(\zeta_1^+ + \zeta_2^-)}, \\
I_2 &= \frac{1}{(\zeta_2^+)^2(\zeta_2^+ + \zeta_1^-)^2(\zeta_2^+ - \zeta_1^+)(\zeta_2^+ + \zeta_2^-)}, \\
I_3 &= \frac{1}{(\zeta_2^+)^2(\zeta_2^+ + \zeta_1^-)(\zeta_2^+ - \zeta_1^+)^2(\zeta_2^+ + \zeta_2^-)}, \\
I_4 &= \frac{-1}{(\zeta_1^+)^2(\zeta_1^+ + \zeta_1^-)(\zeta_1^+ - \zeta_2^+)(\zeta_1^+ + \zeta_2^-)}\left[\frac{2}{\zeta_1^+} + \frac{1}{\zeta_1^+ + \zeta_2^-} + \frac{1}{\zeta_1^+ - \zeta_2^+} + \frac{1}{\zeta_1^+ + \zeta_1^-}\right].
\end{aligned}$$

For the second module, we are instead solving the integral

$$\langle \sigma_2 \rangle_e = 2\int_{-\infty}^{\infty}\int_{-\infty}^{\infty} d\sigma_2 d\sigma_1 \sigma_2(\beta_1\sigma_1 + \beta_2\sigma_2)\Theta(\beta_1\sigma_1 + \beta_2\sigma_2)\rho(\sigma_1|h_1)\rho(\sigma_2|h_2). \tag{S47}$$

The structure of the integral remains the same. To adjust the result we just have β_2^{-1} in front and we replace $\zeta_1^+ \to \zeta_2^+$ and $\zeta_1^- \to \zeta_2^-$. We can use these two results to find the fitness effect in the first environment $\langle s \rangle_e = \beta_1\langle\sigma_1\rangle_e + \beta_2\langle\sigma_2\rangle_e$.

If instead we select in the second environment, we now care about α_1 and α_2. We must redefine our scaling variables using $\zeta_m^\pm = \left(\sqrt{2\nu_0^{-1} + h_m^2} \pm h_m\hat{\alpha}_m\right)/2|\alpha_m|$ and the expectation for the modular outputs when selecting in environment e' take the same form as environment e with this scaling replacement and $\beta \to \alpha$.

Isolated modules

When loci are restricted to participating in only one module we want to evaluate the integral

$$\langle \sigma_1 \rangle_e = 2\beta_1\int_0^\infty d\sigma_1\sigma_1^2 p(\sigma_1|h_1)\left(\frac{2\beta_1\int_0^\infty d\sigma_1\sigma_1 p(\sigma_1|h_1)}{2\beta_1\int_0^\infty d\sigma_1\sigma_1 p(\sigma_1|h_1) + 2\beta_2\int_0^\infty d\sigma_2\sigma_2 p(\sigma_2|h_2)}\right). \tag{S48}$$

This is the expectation of the effect of the mutation given that the locus is in module 1 multiplied by the probability that a mutation will fix in module 1. This tells us that as h_1 increases the rate of fixable mutations in module 1 decreases and it is more likely that module 2 will contain the mutation that fixes. Writing each type of integral out, we have:

$$2\beta_n\int_0^\infty d\sigma_n\sigma_n^2 p(\sigma_n|h_n) = \frac{8\beta_n\nu_0^{-1}}{\sqrt{2\nu_0^{-1} + h_n^2}\left(h_n + \sqrt{2\nu_0^{-1} + h_n^2}\right)^3} = \frac{8\beta_n\nu_0^{-1}}{\zeta_n\left(h_n + \zeta_n\right)^3},$$

$$2\beta_n\int_0^\infty d\sigma_n\sigma_n p(\sigma_n|h_n) = \frac{8\beta_n\nu_0^{-1}}{\sqrt{2\nu_0^{-1} + h_n^2}\left(h_n + \sqrt{2\nu_0^{-1} + h_n^2}\right)^2} = \frac{8\beta_n\nu_0^{-1}}{\zeta_n\left(h_n + \zeta_n\right)^2},$$

where $\zeta_n = \sqrt{2\nu_0^{-1} + h_n^2}$. Therefore we have:

$$\langle \sigma_1 \rangle_e = \frac{8\beta_1\nu_0^{-1}}{\zeta_1\left(h_1 + \zeta_1\right)^3}\left(\frac{8\beta_1\nu_0^{-1}}{\zeta_1\left(h_1 + \zeta_1\right)^2}\right)\left(\frac{8\beta_1\nu_0^{-1}}{\zeta_1\left(h_1 + \zeta_1\right)^2} + \frac{8\beta_2\nu_0^{-1}}{\zeta_2\left(h_2 + \zeta_2\right)^2}\right)^{-1},$$

bioRxiv preprint doi: https://doi.org/10.64898/2026.09.29.755220; this version posted September 30, 2026. The copyright holder for this preprint (which was not certified by peer review) is the author/funder, who has granted bioRxiv a license to display the preprint in perpetuity. It is made available under a CC-BY 4.0 International license.

$$\langle\sigma_2\rangle_e = \frac{8\beta_2\nu_0^{-1}}{\zeta_2\left(h_2+\zeta_2\right)^3}\left(\frac{8\beta_2\nu_0^{-1}}{\zeta_2\left(h_2+\zeta_2\right)^2}\right)\left(\frac{8\beta_1\nu_0^{-1}}{\zeta_2\left(h_1+\zeta_1\right)^2}+\frac{8\beta_2\nu_0^{-1}}{\zeta_2\left(h_2+\zeta_2\right)^2}\right)^{-1},$$

$$\langle\sigma_1\rangle_{e'} = \frac{8\alpha_1\nu_0^{-1}}{\zeta_1\left(h_1+\zeta_1\right)^3}\left(\frac{8\alpha_1\nu_0^{-1}}{\zeta_1\left(h_1+\zeta_1\right)^2}\right)\left(\frac{8\alpha_1\nu_0^{-1}}{\zeta_1\left(h_1+\zeta_1\right)^2}+\frac{8\alpha_2\nu_0^{-1}}{\zeta_2\left(h_2+\zeta_2\right)^2}\right)^{-1},$$

$$\langle\sigma_2\rangle_{e'} = \frac{8\alpha_2\nu_0^{-1}}{\zeta_2\left(h_2+\zeta_2\right)^3}\left(\frac{8\alpha_2\nu_0^{-1}}{\zeta_2\left(h_2+\zeta_2\right)^2}\right)\left(\frac{8\alpha_1\nu_0^{-1}}{\zeta_2\left(h_1+\zeta_1\right)^2}+\frac{8\alpha_2\nu_0^{-1}}{\zeta_2\left(h_2+\zeta_2\right)^2}\right)^{-1}.$$

If we want to find at what values of h_1 and h_2 we will reach steady state, we can ask when the two modules themselves reach steady state. This will occur when

$$\langle\sigma_1\rangle_e = -\langle\sigma_1\rangle_{e'}, \quad \langle\sigma_2\rangle_e = -\langle\sigma_2\rangle_{e'}. \tag{S49}$$

The previous calculation is the result of both environments caring positively about both modules. This creates no tension in the system and we will not observe a steady state – selection will continue to improve both environments.

If instead, an environment cares negatively about one of the modules, the fixation probability will now focus on the deleterious mutations which exist in that module – the side of the DFE which is less than zero. Since the modular DFE is generally non-symmetric (especially when a module is very weakly or strongly adapted), we can not simply apply a negative sign to the integral from 0 to ∞. Instead we we have to perform the integral from $-\infty$ to 0.

If we consider the case where the β vector exists in quadrant I and the α vector is in quadrant III, we have the following calculation for the expectation of the effect on module 2 output when we are selecting in environment 1:

$$I_{1e} = \frac{16\beta_1\nu_0^{-1}}{\zeta_1(\zeta_1+h_1)^3}, \quad S_{1e} = \frac{4\beta_1\nu_0^{-1}}{\zeta_1(\zeta_1+h_1)^2},$$

$$I_{2e} = \frac{16\beta_2\nu_0^{-1}}{\zeta_2(\zeta_2+h_2)^3}, \quad S_{2e} = \frac{4\beta_2\nu_0^{-1}}{\zeta_2(\zeta_2+h_2)^2},$$

$$I_{1e'} = \frac{16\alpha_1\nu_0^{-1}}{\zeta_1(\zeta_1-h_1)^3}, \quad S_{1e'} = \frac{-4\alpha_1\nu_0^{-1}}{\zeta_1(\zeta_1-h_1)^2},$$

$$I_{2e'} = \frac{16\alpha_2\nu_0^{-1}}{\zeta_2(\zeta_2-h_2)^3}, \quad S_{2e'} = \frac{-4\alpha_2\nu_0^{-1}}{\zeta_2(\zeta_2-h_2)^2}.$$

We then have the following expressions for $\langle\sigma_1\rangle_e$, $\langle\sigma_2\rangle_e$, $\langle\sigma_1\rangle_{e'}$, and $\langle\sigma_2\rangle_{e'}$,

$$\langle\sigma_1\rangle_e = I_{1e}\left(\frac{S_{1e}}{S_{1e}+S_{2e}}\right), \quad \langle\sigma_2\rangle_e = I_{2e}\left(\frac{S_{2e}}{S_{1e}+S_{2e}}\right),$$

$$\langle\sigma_1\rangle_{e'} = I_{1e'}\left(\frac{S_{1e'}}{S_{1e'}+S_{2e'}}\right), \quad \langle\sigma_2\rangle_{e'} = I_{2e'}\left(\frac{S_{2e'}}{S_{1e'}+S_{2e'}}\right).$$

Since each locus is isolated to one module, the system can only be put under tension at the level of module dependence. Therefore, a steady state is reached in the system only when each module has opposing dependencies: one environment vector is in QII and the other is in QIV or QI and QIII, respectively.

Homogeneous modules

In the homogeneous modules model, the locus participation is the same for both modules, $\mu_{im} = \mu_i$. We again write out the expectation of the output of module 1 when selecting in environment e as

$$\langle\sigma_1\rangle_e = 2\int_{-\infty}^{\infty}\int_{-\infty}^{\infty} d\sigma_2 d\sigma_1 \sigma_1(\beta_1\sigma_1+\beta_2\sigma_2)\Theta(\beta_1\sigma_1+\beta_2\sigma_2)P(\sigma_1,\sigma_2|h_1,h_2), \tag{S50}$$

where the JDFE is defined by the integral

$$P(\sigma_1,\sigma_2|h_1,h_2) = \int_0^1 d\mu\, P(\nu)\,\frac{1}{4\nu}\varphi\left(\frac{\sigma_1+2\nu h_1}{2\sqrt{\nu}}\right)\varphi\left(\frac{\sigma_2+2\nu h_2}{2\sqrt{\nu}}\right). \tag{S51}$$

We once more assume the distribution of variance fractions is exponential, $P(\nu) = \nu_0^{-1}e^{-\nu/\nu_0}$. Defining the fitness effect in environment e as $s = \beta_1\sigma_1 + \beta_2\sigma_2$ and substituting in the JDFE, Eq. (S50) becomes

$$\langle\sigma_1\rangle_e = 2\int_0^1 d\nu P(\nu)\frac{1}{4\nu}\int_{-\infty}^{\infty}\int_{-\infty}^{\infty} d\sigma_2 d\sigma_1 \sigma_1(s)\Theta(s)\,\varphi\left(\frac{\sigma_1+2\nu h_1}{2\sqrt{\nu}}\right)\varphi\left(\frac{\sigma_2+2\nu h_2}{2\sqrt{\nu}}\right). \tag{S52}$$

bioRxiv preprint doi: https://doi.org/10.64898/2026.09.29.755220; this version posted September 30, 2026. The copyright holder for this preprint (which was not certified by peer review) is the author/funder, who has granted bioRxiv a license to display the preprint in perpetuity. It is made available under a CC-BY 4.0 International license.

Now we can recognize the integrals over σ_1 and σ_2 against the two standard normals can be written as the expectation over the conditional Gaussian pair $\sigma_1, \sigma_2 \mid \mu$ for a fixed value of μ,

$$\langle \sigma_1 \rangle_e = 2 \int_0^1 d\nu \, P(\nu) \, \mathbb{E}_\nu[\sigma_1 s \, \Theta(s)]. \tag{S53}$$

Although σ_1 and σ_2 are conditionally independent Gaussians given μ, the presence of $\Theta(s)$ couples them inside the integrand. So the double integral over σ_1, σ_2 collapses into a single joint expectation, $\mathbb{E}_\nu[\sigma_1 s \, \Theta(s)]$, taken over the pair (σ_1, σ_2). By the law of total expectation, $\mathbb{E}[\sigma_1 s \, \Theta(s)] = \int_0^\infty \mathbb{E}[\sigma_1 \mid s] \, s \, P(s) \, ds$, where $P(s)$ is the marginal density of s given μ. If we define $F = \beta_1 h_1 + \beta_2 h_2$ and $D = \beta_1^2 + \beta_2^2$, then we can write out the means and variances of σ_1 and s as $\mathbb{E}[\sigma_1] = -2\nu h_1$, $\mathbb{E}[s] = -2\nu F$, $\text{Var}(s) = 4\nu D$, and $\text{Cov}(\sigma_1, s) = 4\nu\beta_1$. Using these, the conditional is

$$\mathbb{E}[\sigma_1 | s] = -2\nu h_1 + \frac{\beta_1}{D}(s + 2\nu F) = 2\mu A + Bs, \tag{S54}$$

where $A = F\beta_1/D - h_1$ and $B = \beta_1/D$. With this expression, the expectation in Eq. (S53) can be written as

$$\mathbb{E}[\sigma_1 s \, \Theta(s)] = \int_0^\infty (2\mu A + Bs) \, s \, P(s) \, ds = 2\mu A \int_0^\infty sP(s) \, ds + B \int_0^\infty s^2 P(s) \, ds. \tag{S55}$$

Since $s|\mu \sim \mathcal{N}(-2\nu F, 4\nu D)$, the truncated moments are

$$\int_0^\infty sP(s) \, ds = -2\mu F \, \Phi\left(-F\sqrt{\frac{\nu}{D}}\right) + 2\sqrt{\nu D} \, \phi\left(-F\sqrt{\frac{\nu}{D}}\right), \tag{S56}$$

$$\int_0^\infty s^2 P(s) \, ds = (4\nu^2 F^2 + 4\nu D) \, \Phi\left(-F\sqrt{\frac{\nu}{D}}\right) - 4\nu^{3/2} F\sqrt{D} \, \phi\left(-F\sqrt{\frac{\nu}{D}}\right), \tag{S57}$$

where $\Phi(x)$ and $\phi(x)$ denote the standard normal cumulative distribution function and probability density function, $\Phi(x) = \int_{-\infty}^x dt \, e^{-t^2/2}/\sqrt{2\pi}$ and $\phi(x) = e^{-x^2/2}/\sqrt{2\pi}$, respectively. Substituting these truncated moments into Eq. (S55), and then into Eq. (S53), leaves only integrals over ν. All resulting terms can be expressed as linear combinations of three basic integrals. Defining $r = 1/\mu_0$ and $q = r + F^2/2D$, these integrals are

$$J_1 = \int_0^1 \nu e^{-r\nu} \Phi\left(-F\sqrt{\frac{\nu}{D}}\right) d\nu, \tag{S58}$$

$$J_2 = \int_0^1 \nu^2 e^{-r\nu} \Phi\left(-F\sqrt{\frac{\nu}{D}}\right) d\nu, \tag{S59}$$

$$J_{3/2} = \int_0^1 \nu^{3/2} e^{-r\mu} \phi\left(-F\sqrt{\frac{\nu}{D}}\right) d\nu. \tag{S60}$$

And they evaluate to

$$J_1 = \frac{1}{2r^2} - e^{-r}\left(\frac{1}{r} + \frac{1}{r^2}\right) \Phi\left(-\frac{F}{\sqrt{D}}\right) - \frac{F}{2\sqrt{2\pi D}}\left(\frac{G_{1/2}(q)}{r} + \frac{G_{-1/2}(q)}{r^2}\right), \tag{S61}$$

$$J_2 = \frac{1}{r^3} - e^{-r}\left(\frac{1}{r} + \frac{2}{r^2} + \frac{2}{r^3}\right) \Phi\left(-\frac{F}{\sqrt{D}}\right) - \frac{F}{2\sqrt{2\pi D}}\left(\frac{G_{3/2}(q)}{r} + \frac{2G_{1/2}(q)}{r^2} + \frac{2G_{-1/2}(q)}{r^3}\right), \tag{S62}$$

$$J_{3/2} = \frac{1}{\sqrt{2\pi}} G_{3/2}(q), \tag{S63}$$

where $G_\nu(q) = \int_0^1 \mu^\nu e^{-q\mu} \, d\mu = \gamma(\nu+1, q)/q^{\nu+1} = \Gamma(\nu) - \gamma(\nu, q)$ is the upper incomplete gamma function. Finally we can express the expectation of the modular fitness effect when selecting in environment e in terms of these solutions and previously defined parameters as

$$\langle \sigma_1 \rangle_e = I_1 + I_2 + I_3 + I_4, \tag{S64}$$

where

$$I_1 = -8rFAJ_2, \qquad I_2 = 8rA\sqrt{D} \, J_{3/2}, \tag{S65}$$

$$I_3 = 8rB(F^2 J_2 + DJ_1), \qquad I_4 = -8rBF\sqrt{D} \, J_{3/2}. \tag{S66}$$

For the other three modular expectations the solutions come in similar forms with the defined variables. If instead we are finding the expectation of σ_2, $A \to A' = F\beta_2/D - M_2$ and $B \to B' = \beta_2/D$. And when selecting in environment e' the $\beta's$ are replaced by $\alpha's$.

bioRxiv preprint doi: https://doi.org/10.64898/2026.09.29.755220; this version posted September 30, 2026. The copyright holder for this preprint (which was not certified by peer review) is the author/funder, who has granted bioRxiv a license to display the preprint in perpetuity. It is made available under a CC-BY 4.0 International license.

Experimental Materials and Methods

Growth assay details

Frozen stocks were revived by completely thawing all segregant libraries, mixing well, and transferring 8 µl of frozen stock into 120 µl of YPD+Hyg+Nat (300 µg /ml HygB (Gold Biotechnology Inc #H27010) and 20 µg /ml cloNAT (Gold Biotechnology N-500-100)) in a 96-well flat-bottomed plate. Each segregant was kept separate in a well, and is identified by the well number. These were allowed to grow for 36 hours, shaken at 30°C, until saturation. After this initial grow-up, we added 2 µl of saturated culture to 126 µl of chosen media (YPD, YNB, or Glyc) in flat-bottomed, unshaken 96-well plates, and allowed to saturate in the appropriate temperature. After 24 hours of growth, we marked this as time point zero, sampled for sequencing, and conducted the next transfer. Each segregant occupies the same well in all plates, for all transfers. This $1 : 2^6$ transfer was conducted every 24 hours for 8 days, leading to 42 generations of growth for the assay.

Growth Condition	Details of Media
YPD at 23°C	1% yeast extract, 2% Bacto peptone, grown on bench
YPD at 30°C	1% yeast extract, 2% Bacto peptone, 2% dextrose, grown at 30°C and 40% humidity
YPD at 37°C	1% yeast extract, 2% Bacto peptone, 2% dextrose, grown at 37°C and 50% humidity
Glycerol at 30°C	1% yeast extract, 2% Bacto peptone, 0.5% dextrose, 3% glycerol, grown at 30°C and 40% humidity
YNB at 30°C	Yeast Nitrogen Base w/o amino acids, 2% dextrose, grown at 30°C and 40% humidity

Table S1: Growth Conditions and Media Details. All media also contained 1 µg/ml ampicillin. Reagents used: Yeast Extract (VWR #76628-828), Bacto Peptone (VWR #76628-742), Dextrose (Fisher Sci #BD-215530), Yeast Nitrogen Base (VWR #90004-400), Glycerol (VWR #BDH1172), Ampicillin (Sigma-Aldrich #A9518).

For pelleting and sequencing, we took 45 µl of each segregant and mixed them into a single pellet at each transfer. We could do this since each barcode corresponds to a specific plasmid library (which identifies the segregant) and edge (which identifies the mutation). This allows us to pool before DNA extractions and sequencing, and demultiplex data during analysis. Each pellet was spun down, the supernatant discarded, and the pellet frozen at -20°C until it was prepared for sequencing. Three different replicate assays were conducted for 5 different growth conditions, leading to a total of 15 plates being maintained for each day of the transfer. All 15 replicates were assayed at the same time, though each replicate used a different batch of growth media.

DNA Extractions for barcode sequencing

Each mixed pellet was approximately 4 mL. A full cell pellet was incubated at 37°C in 100 µl zymolyase lysis buffer (5 mg/mL Zymolyase 20T (Nacalai Tesque), 1 M sorbitol (Sigma-Aldrich S1876), 100 mM sodium phosphate pH 7.4, 10 mM EDTA (Thermofisher B49), 0.5% 3-(N,N-Dimethylmyristylammonio)-propanesulfonate (Sigma-Aldrich #40772), 200 µg/̃mL RNAse A (QIAgen #19101), and 20 mM DTT(Goldbio) for 45 minutes. 400 µl of a binding buffer with guanidine thiocyanate (4 volumes of 100 mM MES pH 5 (Goldbio #M-090), 4.125 M guanidine thiocyanate(Goldbio #G-210), 25% isopropanol, and 10 mM EDTA was added, well mixed, and spun down to remove any cell debris. The supernatant was added to a silica column (IBI Scientific #IBI-IB47207) to allow the DNA to bind to the column, and the flowthrough was discarded. After binding, the columns were washed with 400 µl of a first wash buffer (10% guanidine thiocyanate, 25% isopropanol, 10 mM EDTA) and then 600 µl of a second wash buffer (80% ethanol, 10 mM Tris pH 8 (Corning #46-031-CM)). An extra spin down step for 3 minutes allowed any remaining ethanol to evaporate, for better downstream processing. All samples were eluted into 50 µl of molecular-grade water, yielding between 20–30 ng/µl of gDNA, depending on growth conditions.

Sequencing Library Preparation and PCR protocols

For each sample, we performed round 1 reactions which enable each amplicon to be tagged with a UMI. Each forward primer also has a random 7 base-pair region. Each amplicon should have a unique UMI; repeats may occur due to PCR bias and are removed during data analysis. There are also 12 different forward primers to generate an offset in each amplicon, creating the diversity needed for a patterned sequencing flow cell.

To set up the reaction, we combined 15 µl gDNA, 25 µl 2X Kapa HotStart HiFi MM, 3 µl 10 µM TnRS1 primer, 3 µl 10 µM TnFX primer, and 4 µl molecular-grade water. We ran the PCR protocol: 1) 95°C for 3:00, 2) 98°C for 0:20, 3) 60°C for 0:30, 4) 72°C for 0:30, GO TO step 2 for 4 cycles, 5) 72°C for 1:00. This was purified with a left-sided cleanup using Aline magnetic beads (0.85x ratio) and eluted in 30 µl of molecular-grade water. All primers can be found in Johnson et al. [2019] and were ordered from Integrated DNA Technologies (IDT).

To add Illumina adapters, this cleaned-up PCR product was amplified and dual-tagged in round 2 using Nextera primers. The round 2 reaction combined 25 µl purified PCR 1 product, 8 µl molecular-grade water, 10 µl Kapa HiFi Buffer, 1 µl KAPA HiFi HotStart

bioRxiv preprint doi: https://doi.org/10.64898/2026.09.29.755220; this version posted September 30, 2026. The copyright holder for this preprint (which was not certified by peer review) is the author/funder, who has granted bioRxiv a license to display the preprint in perpetuity. It is made available under a CC-BY 4.0 International license.

DNA Polymerase, 1 µl dNTP mix, 2.5 µl 10 µM N7XX primer (Nextera), and 2.5 µl 10 µM S5XX primer (Nextera), and used the PCR protocol: 1) 95°C for 3:00, 2) 98°C for 0:20, 3) 61°C for 0:30, 4) 72°C for 0:30, GO TO step 2 for 25 cycles, 5) 72°C for 2:00. After visualization on an agarose gel, we cleaned the prepped libraries using another left-sided bead cleanup and Aline magnetic beads (0.85x ratio), eluting in 50$\sim$ μl of molecular-grade water.

All libraries were quantified using a fluorescent plate-based assay on the SpectraMax i3 and the AccuGreen^{TM} High Sensitivity dsDNA Quantitation Kit (Biotium #31066). All prepped libraries were pooled by weight. A final 0.8x-0.2x left and right sided cleanup using magnetic Aline beads was performed on the pool to remove any remaining small and large fragments, and fragment size distribution was checked on an Agilent Tapestation 4200. The sequencing was done on 2 separate runs on NovaSeq X Plus 10B lanes (Illumina) using paired end, 2x150 reads.

Inferring mutational fitness from sequencing data

The mutational fitness values were inferred using the process outlined in Johnson et al. [2019]. We measure the log-frequency slope between successive time-points (each time-point must have a minimum of 4000 total counts). Since the neutral barcodes are already known, we can use the median log-frequency slope of the neutral set of barcodes to make the mean fitness correction to these slopes. After excluding assays with less than 5 neutral barcodes (with at least 30 counts each) or less than 3 usable time points, we find fitness effects by scaling values, and averaging over all time intervals that pass our thresholds in the assay.

Since each mutation or "edge" is redundantly barcoded, we can exclude outliers in a group of barcodes for a particular mutation by using a log-likelihood test of belonging to the group. For this process, each group must contain at-least three redundant barcodes. We measure the fitness effect for all barcodes of a mutation, and combine counts from all barcodes that are within a 0.01 of the median fitness effect for that mutation (called the 'reference' for that pool. For a barcode where the fitness effect is further than 0.01 from this median, we use the combined counts and the counts of that particular barcode and find the log-likelihood of 1) barcode and combined 'reference' have the same fitness, and 2) the barcode and combined 'reference' have different fitnesses, modeling non-stochastic changes in frequency of lineages, where the log-likelihood of the data at each time-point t, is given by a frequency prediction $f_{k,t}$ and multinomial number of reads $n_{k,t}$ for each of k lineages. We exclude the barcodes with a LL ratio above a heuristic cutoff, as determined by Johnson et al. [2019].

After exclusion of these outliers, we can further reduce the noise in measurements by collapsing redundant barcodes per mutation into combined barcodes (cBCs). These cBC's have lower per-barcode noise due to having higher counts, but still preserve replicate fitness measurements. We repeat the fitness measurement protocol on these cBCs, and then average to get the fitness effect of a mutation against a particular genetic background. We set a threshold of a minimum number of three cBC's for a mutation to be included. Johnson et al. [2019] have also excluded a few strains and mutations by visual inspection, those are excluded from our analysis too.

Once all three replicate fitness effect values are found we only include mutations which agree among these replicates. We first exclude any mutations which only have measurements in one replicate. For each mutation with two or more available replicate estimates, the maximum pairwise disagreement, $\Delta_{\max} = \max\left(s_{\text{rep1}}, s_{\text{rep2}}, s_{\text{rep3}}\right) - \min\left(s_{\text{rep1}}, s_{\text{rep2}}, s_{\text{rep3}}\right)$, was computed. Mutations with $\Delta_{\max} \leq 0.05$ were considered reproducible and retained, using the average of all available replicates. For mutations with three replicates whose overall disagreement exceeded 0.05, we tested whether any two of the three replicates agreed with each other within 0.05; if so, the mutation was retained using the mean of the two agreeing replicates, with the disagreeing replicate excluded. Mutations for which no pair of replicates agreed within 0.05, or for which only two replicates were available and disagreed beyond 0.05, were excluded from downstream analysis. The 0.05 threshold was chosen based on the empirical distribution of replicate disagreement across mutations (median $\Delta_{\max}$ of 0.01–0.02, with the great majority of mutations disagreeing by less than 0.05), such that the filter removed a minority of poorly-reproducing measurements while retaining the bulk of the dataset.

Background Fitness Estimation

To set up the flow cytometry assays, we revived frozen stocks of the strain backgrounds without any mutations. 2μl of fully thawed culture was added to 126 μl YPD and grown till saturation in shaken, flat bottomed plates. In addition, the florescent reference strain containing a mCitrine marker (LK3-B08 from Jerison et al. [2017] was also revived and grown to saturation in flat bottomed plates. After an initial growup, the reference strain (LKB08) and the parental strains were mixed equiproportionaly, and 2 μl of this was transfered to fresh YPD media. A 6 generation assay (to match the assays for the fitness effects) was conducted by transferring 2 uL of the co-culture to fresh media. The saturated co-culture was the initial time point, and the end point was taken after a further 6 generations. At each time point, 10 uL of saturated culture was transferred to 116 uL of cold, sterile PBS, and kept at 4C before being put through the flow cytometer.

Samples were run on the 4 laser LSR Fortessa and a 5 laser FAC A3 Symphony. All Glycerol and YNB samples (except rows E,F,G,H from YNB Rep1 at timepoint 1) were run on the Symphony, and all other samples (including rows E,F,G,H from YNB Rep1 at timepoint 1) were run on the Fortessa. For each well, 20,000 cells were counted, and the cytometer had a wash step between each well.

For the analysis of flow cytometry data, the first and last 10% of counted events were discarded. Potential doublet events (where the FSC-H to FSC-W ratio was above a threshold of 1.2) were also discarded. The exact ratio used made little difference to our calculations. A Gaussian mixture model was fitted to the data to separate cells into two types (fluorescent reference vs wild type parental strain) and each type was counted separately. Certain counts were manually fixed by eye if the GMM fit was deemed incorrect. Once counted, fitness against the reference florescent strain was measured by calculating $y_0 = \frac{1}{\tau}\ln\left[N_f^{WT} N_i^r / (N_i^{WT} N_f^r)\right]$

bioRxiv preprint doi: https://doi.org/10.64898/2026.09.29.755220; this version posted September 30, 2026. The copyright holder for this preprint (which was not certified by peer review) is the author/funder, who has granted bioRxiv a license to display the preprint in perpetuity. It is made available under a CC-BY 4.0 International license.

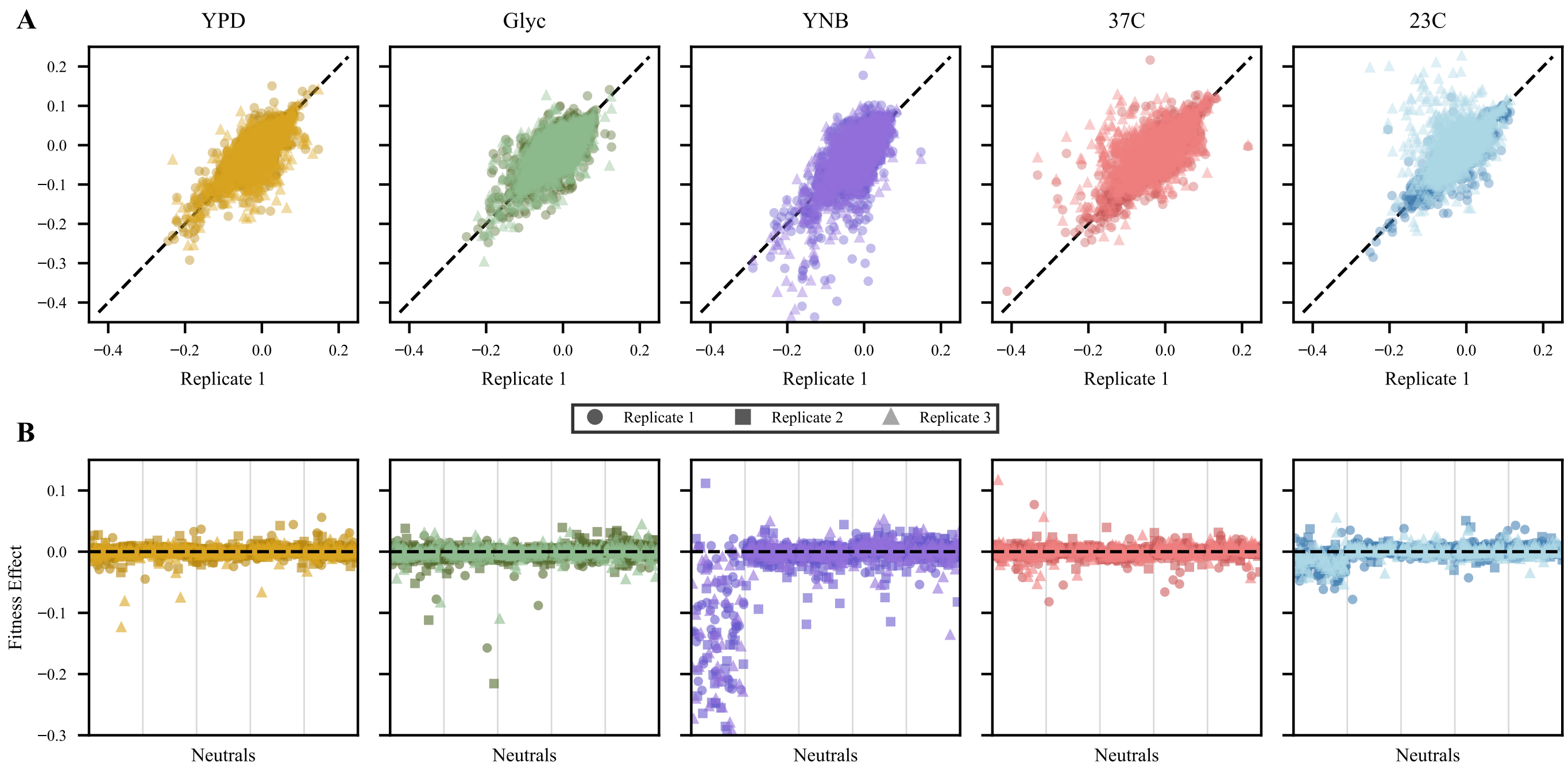

Figure S10: **Replicate – replicate comparisons and neutral analysis of mutational fitness effects. (A)** Mutational fitness measured in Replicate 1 vs. Replicate 2 (darker squares) and 3 (lighter triangles). **(B)** Mutational fitness measurements for the five marked neutrals in all three replicates. Each replicate can be differentiated by the color and shape of the markers. The first neutral in YNB exhibited a large spread of fitness effects and for this reason was discarded as a neutral.

where $\tau = 6$ and values in the natural log are final and initial counts of the reference and WT strains. For YNB, a 12 generation assay was conducted, and hence $\tau_{YNB} = 12$.

For each background and environment, the difference between replicate fitness estimates was computed, $\Delta_e = y_{e,\text{rep1}} - y_{e,\text{rep2}}$, and the standard deviation of this difference, σ_Δ, was calculated across all backgrounds within that environment. Background-replicate pairs whose absolute difference exceeded two standard deviations of the pooled replicate-difference distribution, $|\Delta_e| > 2\sigma_\Delta$, were considered technical outliers and excluded from further analysis. For all retained background-replicate pairs, the final fitness estimate was recalculated as the mean of the two replicates following outlier removal. We also limit all environments to background fitness values between -0.3 and 0.3 which removes at most three data points per environment and the $37°C$ environment to points above -0.08 due to mutations made in KRE33, a gene involved in small ribosomal subunit assembly, having outsized effects important to this environment [Jerison et al., 2017]. The background fitness values have good inter-replicate correlations and can be found in Fig. S11.

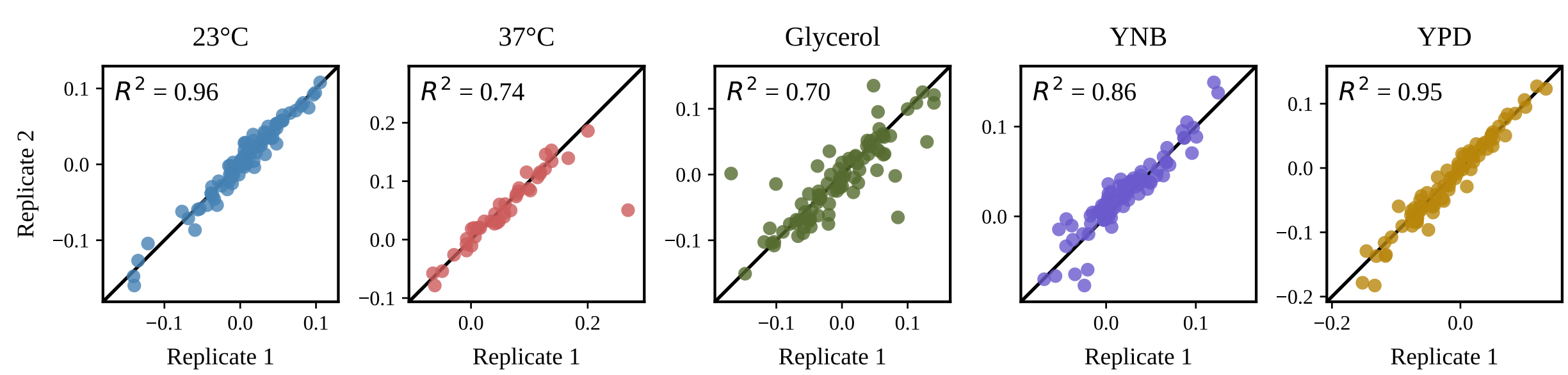

Figure S11: **Background fitness replicate – replicate comparisons.** Inter-replicate correlations of background fitness measurements through flow cytometry after outlier exclusion for all five environments. Replicate-replicate environmental correlations before outlier exclusion are included in data parsing scripts (see Code and Data Availability).
